# Stress distribution in the closure of anterior maxillary diastemas using different restorative approaches: a finite element analysis

**DOI:** 10.1007/s00784-025-06258-9

**Published:** 2025-03-07

**Authors:** Kevser Karakaya, Rahime Zeynep Erdem

**Affiliations:** 1 Department of Prosthodontics, Faculty of Dentistry, Afyonkarahisar Health Sciences University, Afyonkarahisar, Turkey; 2 Department of Restorative, Faculty of Dentistry, Afyonkarahisar Health Sciences University, Afyonkarahisar, Turkey

**Keywords:** CAD-CAM materials, Diastema, Finite element analysis, Full crown, Laminate veneer

## Abstract

**Objective:**

This finite element analysis study aims to evaluate the effects of restoring 1 mm and 2 mm diastemas in maxillary anterior incisors with different CAD-CAM materials on stress distribution and to compare full crown and laminate veneer restorations.

**Materials and methods:**

Maxillary anterior tooth models simulating laminate veneer and full crown restorations were created. The experimental groups were categorized into four based on diastema width (1 mm and 2 mm) and CAD-CAM materials. Loads of 50 N, 150 N, and 250 N were applied vertically at 0° and obliquely at 30° and 60° angles to the central incisor’s incisal midpoint. Von Mises stress values were analyzed numerically and visually with color-coded comparisons for all models.

**Results:**

When laminate veneer restorations were used to close diastemas, the highest stress values under a 250 N vertical load and 2 mm diastema were observed in the lateral incisor. IPS E.max exhibited a stress value of 166.88 MPa for the superstructure, while Lava Ultimate recorded 35.49 MPa in the lateral incisor. Within the dentin, the highest stress was 45.92 MPa for IPS E.max and 65.74 MPa for Lava Ultimate in the lateral incisor. When full crown restorations were employed, the highest stress values under a 250 N vertical load were again recorded in the lateral incisor. IPS E.max showed a stress value of 198.03 MPa for the superstructure, while Lava Ultimate demonstrated 40.80 MPa. The highest stress values within the dentin were 65.20 MPa for IPS E.max and 65.74 MPa for Lava Ultimate in the lateral incisor.

**Conclusions:**

This finite element analysis highlights that the type of restoration and diastema width significantly affect stress distribution in anterior teeth. IPS E.max material exhibited higher stress values, leading to more significant stress accumulation than other materials in laminate veneer and full crown restorations. Additionally, Lava Ultimate showed the highest stress values within the dentin. These findings underscore the importance of selecting appropriate CAD-CAM materials and restoration types to optimize stress distribution in diastema closures, providing critical insights for restoration planning.

## Introduction

Diastema restoration, particularly in the anterior region, represents a critical focus of aesthetic and functional dentistry. A diastema, an interdental space of 0.5 mm or greater, is most commonly observed in the anterior maxillary teeth [1]. While some individuals view a diastema as a characteristic feature, many perceive it as an aesthetic concern that disrupts the symmetry of their smile and may draw undesired attention [2]. Addressing diastema involves various treatment modalities, including orthodontic therapy, restorative approaches, and fixed prosthodontics. Although orthodontic treatment offers a conservative solution, its lengthy duration, appliance visibility, and risk of relapse often prompt patients to seek alternative restorative options [3].

Laminate veneers have gained popularity as a minimally invasive and highly aesthetic solution for diastema restoration [4]. However, in cases of broader diastema or when additional structural reinforcement is required, full crowns may be preferred, offering more excellent durability at the expense of more extensive tooth preparation [5]. Computer-aided design/computer-aided manufacturing (CAD-CAM) technologies have further enhanced the precision, efficiency, and customization of laminate veneers and full crowns [5]. Among CAD-CAM materials, lithium disilicate ceramics, such as IPS e.max CAD (Ivoclar Vivadent AG, Liechtenstein), are highly regarded for their superior fracture resistance, optical properties, and long-term clinical performance [6]. Resin nanoceramics, such as Lava™ Ultimate (3 M ESPE, St. Paul, USA), provide distinct benefits, including improved flexibility, fracture toughness, and ease of intraoral repair [7]. Despite these advances, the differing mechanical properties of these materials underscore the importance of careful selection for optimal clinical outcomes.

Finite element analysis (FEA) has emerged as a powerful tool in dental biomechanics, providing detailed insights into stress distribution within restorations and surrounding dental structures [8]. Stress concentration is particularly significant in diastema restoration, as forces are often concentrated around the interdental space [9]. The thickness of the restorative material, its inherent mechanical properties, and the applied loading conditions critically influence the longevity of laminate veneers and full crowns [9]. Vertical forces typically generate compressive stresses that elevate fracture risk, whereas oblique forces produce complex stress distributions that challenge both the restoration and the underlying tooth structure [10].

Despite advancements in restorative materials and CAD-CAM technology, limited studies have directly compared the biomechanical performance of laminate veneers and full crowns in diastema restoration, particularly under varying widths and loading conditions [11]. This study addresses this gap by systematically analyzing the performance of lithium disilicate and resin nanoceramics in laminate veneers and full crowns to restore 1 mm and 2 mm diastemas.

The findings of this study aim to provide clinically relevant insights to guide material selection and restoration design in diastema cases, thereby contributing to evidence-based dental practice. The null hypothesis asserts that the type of restoration, material choice, and diastema width do not significantly affect stress distribution in restorations or the underlying tooth structures.

## Materials and methods

This study was approved by the Clinical Research Ethics Committee of the Faculty of Dentistry at Afyonkarahisar Health Sciences University on July 7, 2023, under protocol number 2011-KAEK-2. The research was conducted in collaboration with the Faculty of Dentistry at Afyonkarahisar Health Sciences University and Ay Tasarım Ltd. Şti.

Finite element analysis (FEA) was performed to simulate anterior diastema closure under varying conditions, including two diastema widths (1 mm and 2 mm) and two restorative materials (Lava Ultimate and IPS E.max). Eight experimental groups were established by combining diastema widths, restoration types (laminate veneer or full crown), and material choices.

### Model creation

A three-dimensional (3D) geometric model of a maxillary central incisor was developed, including internal anatomical structures such as cortical and spongy bone, the periodontal ligament (PDL), pulp, adhesive layer, and cement. The model was created in STL format and imported into Rhinoceros 4.0 software (3670 Woodland Park Ave N, Seattle, WA, USA). Boolean operations integrated the tooth, PDL, and bone geometries. The periodontal ligament was modeled as a uniform 0.2 mm layer surrounding the root surface using Hypermesh software (Altair Engineering Inc., Detroit, MI, USA). The cortical bone was defined with an external thickness of 2 mm. An adhesive layer (50 μm) and cement layer (100 μm) were added to the tooth surface using the offset command in HyperMesh, and these layers were aligned along the x, y, and z axes to form the outer laminate veneer layer. Completed geometries were transferred to Algor Fempro software (Algor Inc., USA), ensuring spatial accuracy.

### Restoration design

**Fig. 1:**
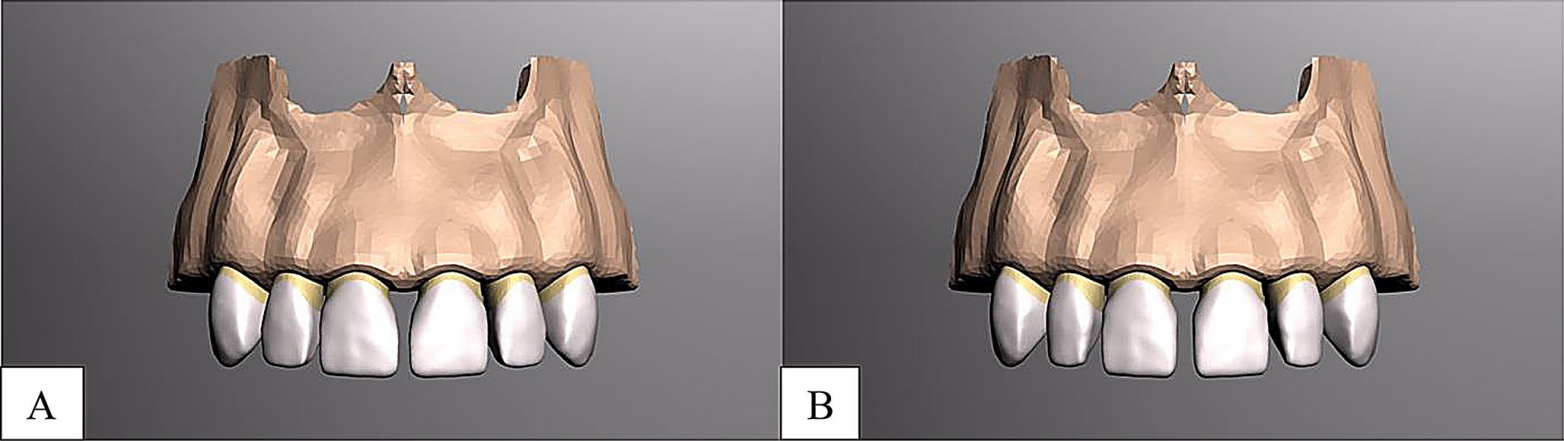
Maxillary anterior tooth modeling. **A**, 1 mm diastema, **B**, 2 mm diastema

**Fig. 2:**
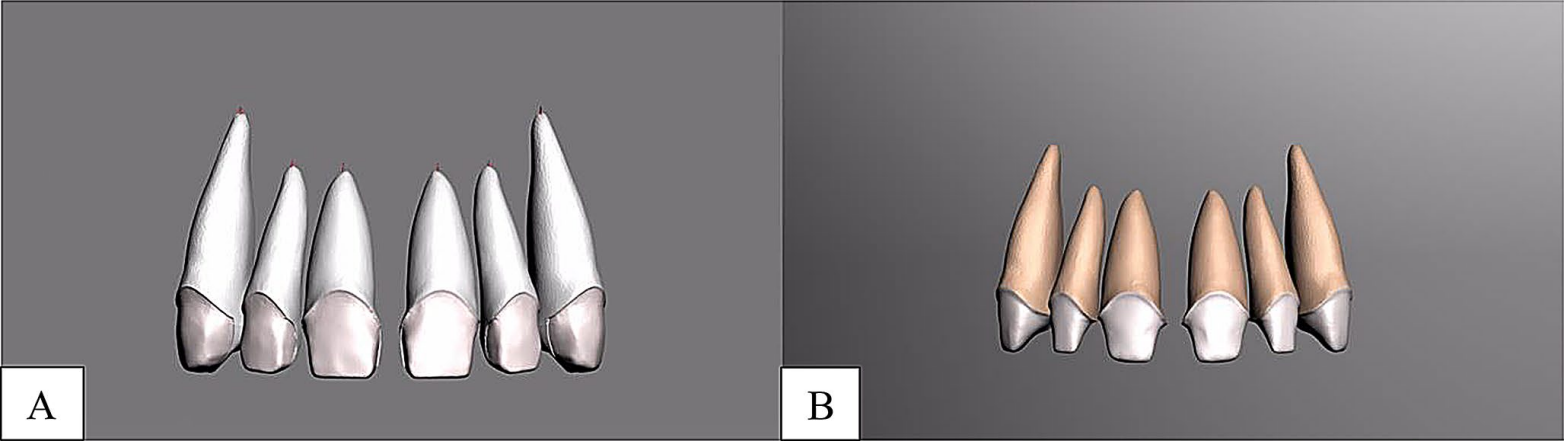
Maxillary anterior tooth modeling **A**, laminate veneer tooth preparatıon, **B**, full crown tooth preparation

**Fig. 3:**
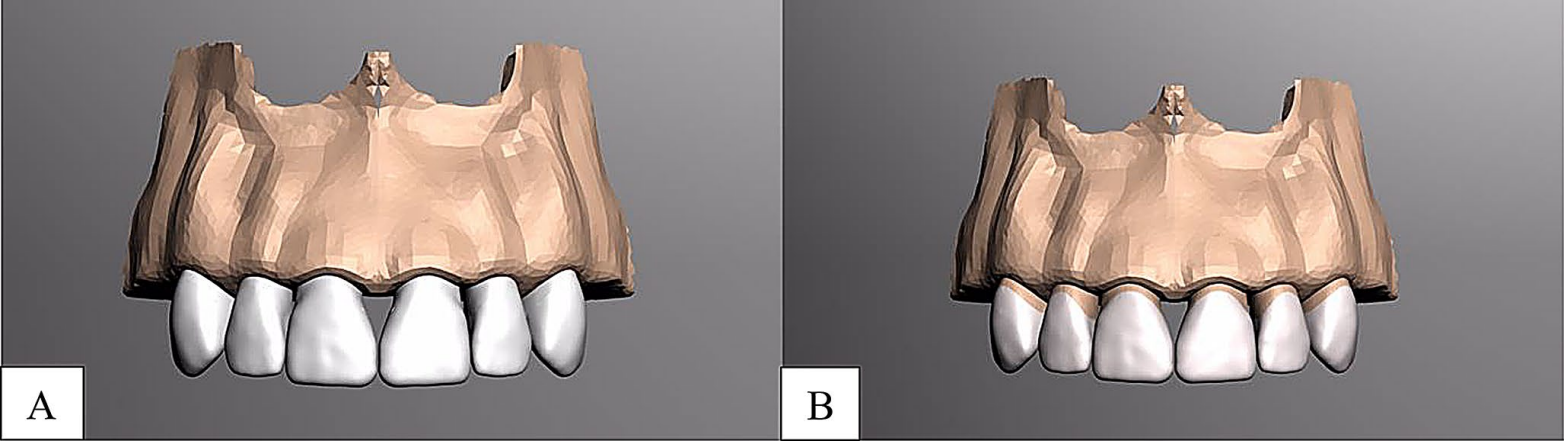
Maxillary anterior tooth modeling **A**, laminate veneer tooth restoration **B**, full crown tooth preparation

Laminate veneer restorations featured a 0.5 mm subgingival chamfer margin, 2 mm incisal reduction, 1.5 mm buccal reduction, and 1.0 mm interproximal and lingual reduction. Full crown restorations were designed with comparable reduction parameters. CAD-CAM materials included Lava Ultimate (3 M ESPE, St. Paul, USA) and IPS E.max CAD (Ivoclar Vivadent AG, Liechtenstein) (Fig. 1, 2, 3).

### Finite element mesh

The 3D geometries were refined and converted into STL format using VRMesh software. These files were imported into Algor Fempro for meshing, where 8-node brick elements formed the primary mesh and 4-node tetrahedral elements were utilized in complex regions to enhance accuracy and flexibility. Linear elements were avoided to preserve structural integrity and ensure reliable results. The models were considered homogeneous, isotropic, and linearly elastic, with mechanical properties (elastic modulus and Poisson’s ratio) assigned based on values from the literature (Table 1). The number of elements and nodes used in the study is presented in Table 2.

**Table 1:** Young’s modulus and Poisson’s ratios of the materials used

	Poisson Ratio	Young Modulus(MPa)	References
Enamel	0.33	84,100	[20]
Dentine	0.32	18,600	[21]
Cement	0.30	18,700	[22]
Periodontal Ligament	0.45	68.9	[17]
Sponge Bone	0.30	1370	[17]
Cortical Bone	0.30	13,700	[17]
Adhesıve	0.29	5000	[23]
Adhesive cement (CLEARFİL ESTHETİC CEMENT)	0.24	9300	[20]
Ceramic (IPS E MAX)	0.23	95,000	Ivoclar vivadent AG, Schaan, Liechtenstein
Composite (LAVA ULTİMATE)	0.45	12,700	[24]

**Table 2:** Number of elements and nodes when 1 mm and 2 mm diastema

		Element	Nodes
Laminate veneer	1 mm diastema	831,803	175,746
	2 mm diastema	835,527	176,093
Full crown	1 mm diastema	1,098,082	212,002
	1 mm diastema	1,098,847	210,650

### Experimental groups

Eight experimental groups were defined by combining two diastema widths (1 mm and 2 mm), two restoration types (laminate veneer and full crown), and two restorative materials (Lava Ultimate and IPS E.max) (Table 3).

**Table 3:** Study groups

Group	Diastema Width	Restoration Type	Material Used
1	1 mm	Laminate Veneer	Lava Ultimate
2	1 mm	Laminate Veneer	IPS E.max
3	2 mm	Laminate Veneer	Lava Ultimate
4	2 mm	Laminate Veneer	IPS E.max
5	1 mm	Full Crown	Lava Ultimate
6	1 mm	Full Crown	IPS E.max
7	2 mm	Full Crown	Lava Ultimate
8	2 mm	Full Crown	IPS E.max

### Loading and analysis

Uniform loads of 50 N, 150 N, and 250 N were applied at the incisal midpoint of the maxillary central incisor, lateral incisor, and canine teeth in a buccolingual direction at vertical (0°) and oblique angles (30° and 60°). Stress distribution within the restorations and supporting structures was evaluated using Von Mises stress criteria, presented numerically and with color-coded visualizations.

### Validation of the FEA model

To enhance the accuracy of the FEA model, its validity was assessed by comparing stress distribution patterns and load transfer mechanisms with findings from previous experimental studies. Specifically, FEA validation protocols commonly used in prosthetic dentistry were considered, and the predicted stress distribution was compared with empirical data from in vitro studies [12, 13].

Material properties and boundary conditions were established for validation based on widely accepted references in prosthetic dentistry (Table 1). A mesh convergence analysis was also performed to ensure that mesh refinement did not significantly alter the stress distribution results. The effect of mesh density on stress distribution was analyzed, and the final mesh size was determined based on convergence criteria to optimize accuracy [14]. This validation process ensured that our FEA model produced biomechanically reliable and clinically relevant results.

### Boundary conditions

**Fig. 4:**
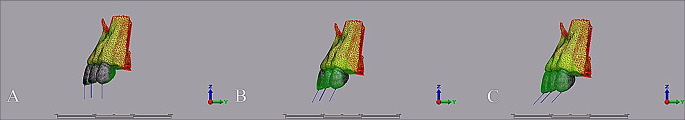
Application of force. **A** vertical force and boundary conditions, **B**, 30^0^ oblique force and boundary conditions, C, 60^0^ oblique force and boundary conditions

The superior surface of the maxilla was constrained to simulate fixation to the skull base, restricting motion to six degrees of freedom. Bonded contact models were applied at interfaces between the trabecular-cortical bone, bone-tooth, and restoration layers to prevent separation or sliding during loading (Fig. 4).

Since the results of finite element stress analyses are based on mathematical calculations and do not involve variance, statistical analysis was not performed. Instead, the emphasis was placed on a detailed evaluation and interpretation of the cross-sectional images, focusing on the magnitude and distribution of stress at the nodes. This study examines the stress in the laminate veneer restoration and tooth structure when different vertical and oblique forces are applied to maxillary anterior teeth with two different diastema widths using two different laminate veneer materials.

## Result

This study aims to assess the biomechanical performance of laminate veneer and full crown restorations under varying diastema widths and restorative materials and offer evidence-based recommendations for clinical applications.

This study evaluated von Mises stress distribution in maxillary central, lateral, and canine teeth restored with Lava Ultimate and IPS E.max materials under varying loading conditions (50 N, 150 N, 250 N) and angles (0°, 30°, 60°). The analysis considered stress distribution in both the superstructure and dentin for laminate veneers and full crowns, with diastema widths of 1 mm and 2 mm (Tables 4, 5, 6 and 7).

**Table 4:** Von mises stress values occurring in the central, lateral, canine teeth superstructure when vertical and oblique forces are applied laminate veneer

Mpa		50 *N*			150 *N*			250 *N*		
Central		0^0^	30^0^	60^0^	0^0^	30^0^	60^0^	0^0^	30^0^	60^0^
Lava Ultimate	1 mm	4.67	1.16	1.93	13.02	3.48	6.58	**22.40**	5.81	9.67
Lava Ultimate	2 mm	4.39	1.54	3.65	14.87	6.34	10.96	**23.74**	7.70	18.27
Ips E max	1 mm	18.22	5.33	11.06	63.44	10.78	28.12	**105.73**	30.15	43.22
Ips E max	2 mm	21.96	7.63	15.47	54.65	27.00	50.27	**109.81**	35.36	61.44
Lateral										
Lava Ultimate	1 mm	7.57	2.58	4.11	22.72	10.00	12.33	**34.81**	12.93	21.95
Lava Ultimate	2 mm	6.91	1.67	5.66	21.29	8.31	11.11	**35.49**	7.16	15.30
Ips E max	1 mm	33.04	22.38	47.64	99.14	109.38	128.31	**139.00**	111.94	114.80
Ips E max	2 mm	27.09	7.55	30.30	81.29	22.65	89.72	**166.88**	32.19	149.53
Canine										
Lava Ultimate	1 mm	3.00	1.65	2.03	9.01	4.96	5.66	**15.03**	8.27	10.48
Lava Ultimate	2 mm	3.32	1.59	2.44	9.96	4.94	9.48	**16.60**	9.97	13.13
Ips E max	1 mm	14.07	15.69	21.56	42.22	47.07	64.68	70.36	**78.46**	59.62
Ips E max	2 mm	14.17	11.79	16.98	42.52	24.01	34.69	**70.86**	40.03	58.06

**Table 5:** Von mises stress values occurring in the central, lateral, canine teeth dentin when vertical and oblique forces are applied laminate veneer

Mpa		50 *N*			150 *N*			250 *N*		
Central		0^0^	30^0^	60^0^	0^0^	30^0^	60^0^	0^0^	30^0^	60^0^
Lava Ultimate	1 mm	9.41	2.27	7.80	27.46	6.83	23.442	**47.08**	11.38	39.02
Lava Ultimate	2 mm	8.67	2.41	9.14	24.69	7.49	27.44	**43.39**	12.08	45.74
Ips E max	1 mm	9.33	2.37	7.69	27.99	7.12	23.07	**45.90**	10.42	38.46
Ips E max	2 mm	8.66	2.49	8.98	27.55	7.70	26.95	**45.92**	12.74	39.08
Lateral										
Lava Ultimate	1 mm	12.61	5.87	10.41	37.85	19.05	31.25	**64.29**	29.39	52.09
Lava Ultimate	2 mm	13.14	5.92	10.17	38.47	17.78	30.52	**65.74**	29.64	50.87
Ips E max	1 mm	12.73	6.32	10.20	36.70	17.55	30.61	**65.43**	28.34	54.71
Ips E max	2 mm	13.64	5.88	9.94	40.94	17.66	29.83	**68.23**	28.38	49.73
Canine										
Lava Ultimate	1 mm	7.00	2.82	5.60	18.42	8.47	16.80	**30.71**	14.11	27.46
Lava Ultimate	2 mm	5.87	2.81	5.86	17.62	8.55	17.42	**29.26**	14.26	29.04
Ips E max	1 mm	6.92	2.80	5.38	20.76	8.18	15.58	**28.94**	12.86	26.16
Ips E max	2 mm	5.97	2.61	5.68	17.93	8.57	15.40	**29.80**	13.05	28.42

**Table 6:** Von mises stress values occurring in the central, lateral, canine teeth superstructure when vertical and oblique forces are applied to full crown

Mpa		50 *N*			150 *N*			250 *N*		
Central		0^0^	30^0^	60^0^	0^0^	30^0^	60^0^	0^0^	30^0^	60^0^
Lava Ultimate	1 mm	2.02	1.15	2.52	8.73	4.23	7.57	**17.29**	6.90	12.61
Lava Ultimate	2 mm	3.17	1.25	2.58	15.00	4.17	7.72	**27.71**	7.55	12.72
IPS E Max	1 mm	8.41	4.94	7.58	23.83	9.01	21.99	**47.24**	22.37	47.24
IPS E Max	2 mm	10.48	2.82	13.20	68.63	8.17	46.76	**114.38**	14.10	81.72
Lateral										
Lava Ultimate	1 mm	8.67	5.67	3.58	83.77	17.03	10.98	**43.37**	28.39	18.31
Lava Ultimate	2 mm	8.16	4.37	4.99	24.48	14.47	14.98	**40.80**	24.11	24.98
IPS E Max	1 mm	32.04	8.82	5.04	131.97	26.47	24.27	**55.94**	44.12	55.94
IPS E Max	2 mm	29.04	12.69	16.65	118.82	44.68	49.97	**198.03**	74.47	83.28
Canine										
Lava Ultimate	1 mm	3.40	0.96	2.79	16.31	3.12	8.37	**27.19**	4.81	10.48
Lava Ultimate	2 mm	1.28	1.75	2.49	13.03	4.63	7.14	**21.72**	8.70	11.01
IPS E Max	1 mm	9.83	3.75	8.65	29.51	9.50	23.03	**27.83**	16.64	27.83
IPS E Max	2 mm	19.11	4.02	7.01	81.82	14.32	25.53	**95.56**	29.98	45.27

**Table 7:** Von mises stress values occurring in the central, lateral, canine teeth dentine when vertical and oblique forces are applied to full crown

Mpa		50 *N*			150 *N*			250 *N*		
Central		0^0^	30^0^	60^0^	0^0^	30^0^	60^0^	0^0^	30^0^	60^0^
Lava Ultimate	1 mm	8.83	1.97	9.94	27.67	5.92	22.41	**44.16**	9.87	37.36
Lava Ultimate	2 mm	9.35	2.41	8.12	26.21	5.90	27.44	**46.79**	12.08	45.74
Ips E max	1 mm	9.61	2.06	9.05	27.68	6.13	26.42	**48.09**	10.19	46.18
Ips E max	2 mm	9.05	2.22	7.60	26.69	6.66	22.82	**46.94**	11.10	38.04
Lateral										
Lava Ultimate	1 mm	13.07	5.85	10.45	39.23	17.55	34.37	**65.38**	29.26	57.29
Lava Ultimate	2 mm	13.82	6.00	10.17	41.48	14.82	30.52	**69.14**	29.64	50.87
Ips E max	1 mm	12.94	5.84	10.70	39.44	17.54	33.21	**63.78**	29.95	55.35
Ips E max	2 mm	13.89	5.88	10.54	38.85	17.64	32.17	**65.20**	29.94	53.62
Canine										
Lava Ultimate	1 mm	7.82	2.01	5.11	23.46	6.05	15.33	**39.10**	10.09	25.33
Lava Ultimate	2 mm	7.54	2.94	5.04	22.63	8.83	17.42	**37.72**	14.26	29.04
Ips E max	1 mm	8.04	2.04	5.19	21.04	6.14	14.78	**40.21**	10.12	26.09
Ips E max	2 mm	7.73	2.50	5.13	23.21	6.91	15.09	**38.69**	12.19	25.16

**Fig. 5:**
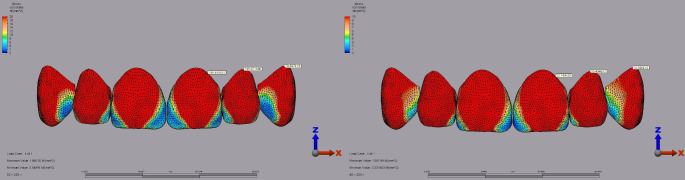
Fig 5.Von Mises stress distribution inthe superstructure of laminate veneer restorations under a 250 N vertical load with a 2 mm diastema for the central, lateral, and canine teeth. **A:** IPS E.max. **B:** Lava Ultimate.

For laminate veneer restorations, the highest superstructure stress in the central incisor was observed with IPS E.max at 109.81 MPa under a 250 N vertical load with a 2 mm diastema. In comparison, Lava Ultimate recorded a lower maximum stress of 23.74 MPa under the same conditions (Fig. 5). In the lateral incisor, IPS E.max demonstrated the highest stress at 166.88 MPa under a 250 N vertical load with a 2 mm diastema, compared to Lava Ultimate, which showed a maximum stress of 35.49 MPa (Fig. 5). In the canine tooth, IPS E.max reached a peak stress of 70.86 MPa under a 250 N vertical load with a 2 mm diastema, while Lava Ultimate recorded 16.60 MPa under identical conditions (Fig. 5).

**Fig. 6:**
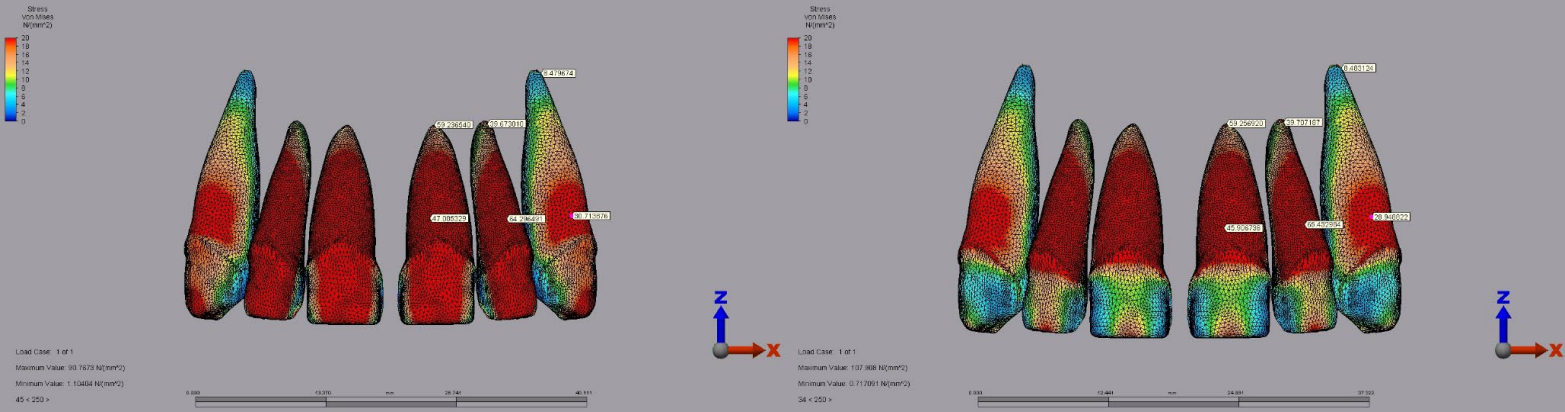
Von Mises stress distribution in the dentin for laminate veneer restorations under a 250 N vertical load with a 1 mm diastema for the central, lateral, and canine teeth. **A**: Lava Ultimate. **B**: IPS E.max

**Fig. 7:**
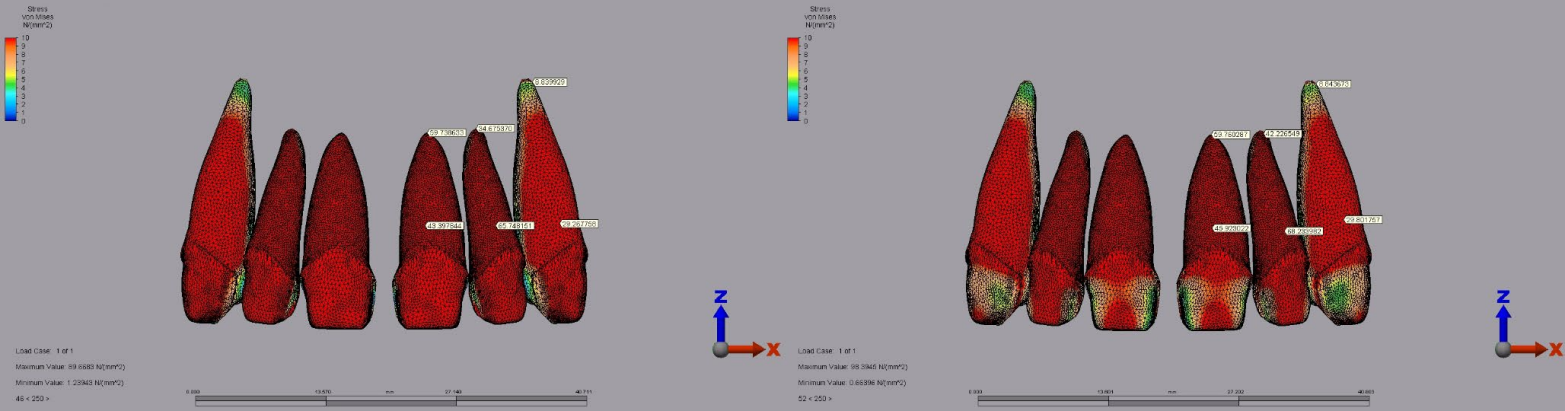
Von Mises stress distribution in the dentin for laminate veneer restorations under a 250 N vertical load with a 2 mm diastema for the central, lateral, and canine teeth. **A**: Lava Ultimate. **B**: IPS E.max

Regarding dentin stress for laminate veneer restorations, the central incisor showed the highest stress of 47.08 MPa with Lava Ultimate under a 250 N load applied vertically with a 1 mm diastema. IPS E.max recorded 45.90 MPa under the same conditions (Fig. 6). For the lateral incisor, the maximum dentin stress was 65.74 MPa with Lava Ultimate and 68.23 MPa with IPS E.max, both under a 250 N vertical load with a 2 mm diastema (Fig. 7). The highest dentin stress with Lava Ultimate in the canine tooth was 30.71 MPa under a 250 N load applied vertically with a 1 mm diastema. IPS E.max recorded 28.94 MPa under similar conditions (Fig. 6).

**Fig. 8:**
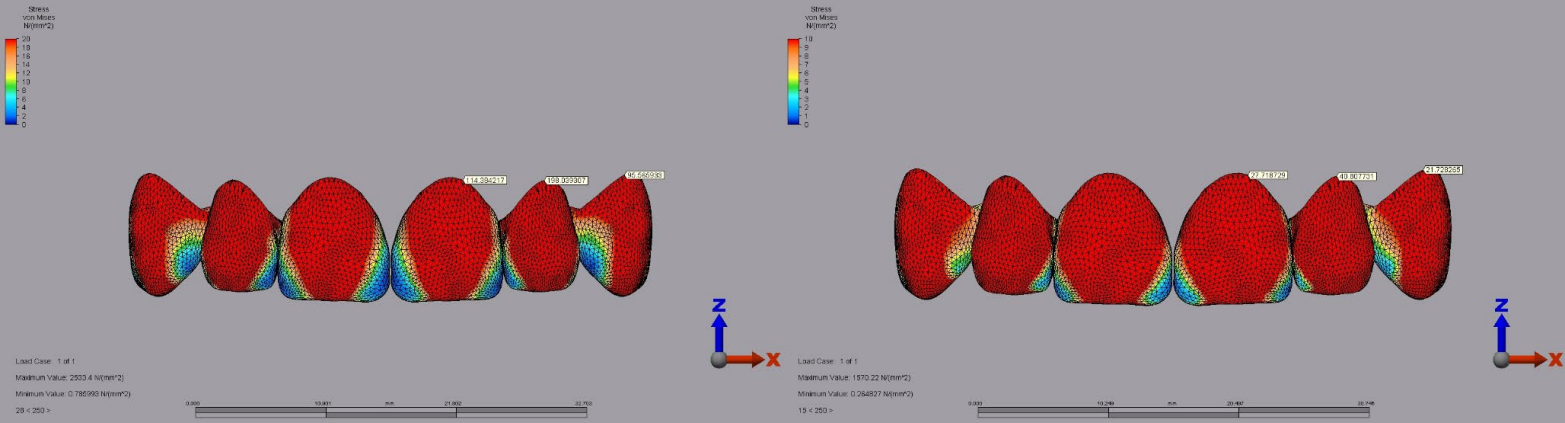
Von Mises stress distribution in the superstructure of full-crown restorations under a 250 N vertical load with a 2 mm diastema for the central, lateral, and canine teeth. **A**: IPS E.max. **B**: Lava Ultimate

IPS E.max exhibited the highest superstructure stress of 114.38 MPa in the central incisor under a 250 N vertical load with a 2 mm diastema for full crown restorations. Lava Ultimate recorded 27.71 MPa under the same conditions (Fig. 8). In the lateral incisor, IPS E.max reached 198.03 MPa under a 250 N vertical load with a 2 mm diastema, significantly higher than Lava Ultimate’s 40.80 MPa under similar conditions (Fig. 8). For the canine tooth, IPS E.max showed a peak stress of 95.56 MPa under a 250 N vertical load with a 2 mm diastema, compared to Lava Ultimate’s 21.72 MPa (Fig. 8).

**Fig. 9:**
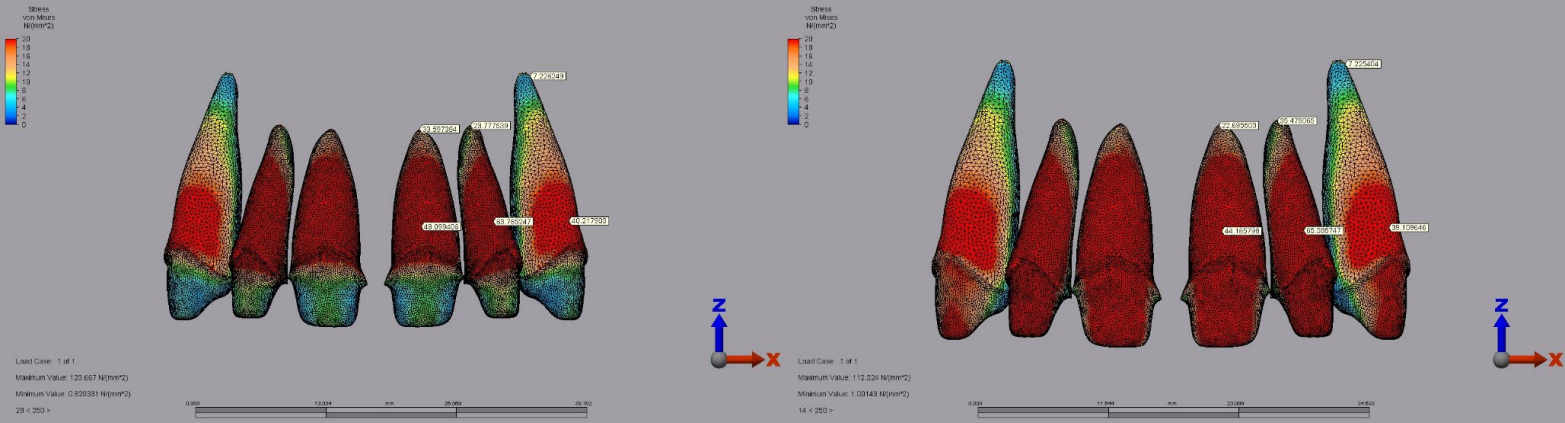
Fig 9.Von Mises stress distribution in the dentin for full crown restorations under a 250 N vertical load with a 1 mm diastema for the central, lateral, and canine teeth. **A:** IPS E.max. **B:** Lava Ultimate.

**Fig. 10:**
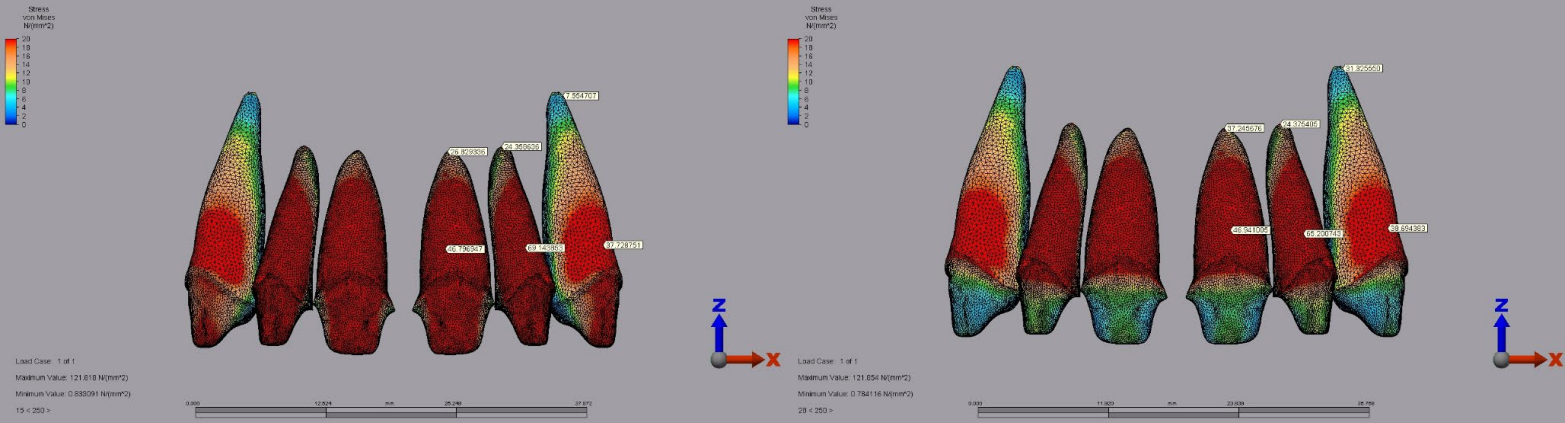
Von Mises stress distribution in the dentin for full crown restorations under a 250 N vertical load wit a 2 mm diastema for the central, lateral, and canine teeth. **A**: Lava Ultimate** B**: IPS E.max.

Dentin stress results for full crown restorations revealed that in the central incisor, the highest stress was 48.09 MPa with IPS E.max under a 250 N load applied vertically with a 1 mm diastema. Lava Ultimate showed 44.16 MPa under the same conditions (Fig. 9). In the lateral incisor, the highest dentin stress was 69.14 MPa with IPS E.max under a 250 N vertical load with a 2 mm diastema. In contrast, Lava Ultimate recorded 65.20 MPa under a 250 N vertical load with a 2 mm diastema (Fig. 10). For the canine tooth, the maximum dentin stress was 39.10 MPa with Lava Ultimate and 40.21 MPa with IPS E.max under a 250 N vertical with a 1 mm diastema, respectively (Fig. 9).

These findings indicate that IPS E.max generally exhibited higher stress values in the superstructure, while Lava Ultimate showed higher stress values in the dentin under certain conditions. The results emphasize the importance of material selection and restoration design in diastema closure to optimize stress distribution and long-term clinical performance.

## Discussion

This study evaluated the stress distribution of various CAD-CAM restorative materials used in maxillary anterior diastema closures and compared laminate veneer and full crown restorations under different loading conditions. Significant differences in stress distribution were observed based on material type, restoration design, and loading direction, leading to the rejection of the null hypothesis.

The FEA model used in this study was compared with previous studies in restorative dentistry, and the consistency of the results was evaluated. The fact that the model outcomes align with prior in vitro and clinical data supports the validity of our approach [12, 13]. However, since direct experimental validation was not performed, future research should incorporate in vitro mechanical tests to further confirm these findings. The FEA model used in this study was validated through comparisons with previous biomechanical studies, demonstrating consistency in stress distribution. However, this model has not undergone direct experimental validation.

Future studies should validate the model results through direct in vitro mechanical testing and patient-specific biomechanical analyses to further assess the reliability of FEA analyses in clinical applications.

Testing load transfer mechanisms in laboratory settings will reinforce FEA’s role as a predictive tool in prosthetic dentistry. This highlights that FEA analyses are powerful predictive tools for clinical applications but may not fully replicate actual clinical conditions.”

### Material properties and stress distribution

The findings of this study align with those of Lai et al. [15], who investigated the biomechanical behavior of lithium disilicate (IPS E.max) and resin nanoceramic (Lava Ultimate) restorations under different endodontic access preparations. Lithium disilicate restorations exhibited higher stress concentrations due to their high elastic modulus, which, while beneficial for structural rigidity, increases the risk of fracture by promoting localized stress accumulation. Conversely, Lava Ultimate demonstrated a more even stress distribution, attributed to its lower elastic modulus, which helps mitigate stress intensity. Similarly, in this study, IPS E.max restorations showed higher stress under vertical loads, whereas Lava Ultimate exhibited lower maximum stress values. These findings suggest that Lava Ultimate may be more appropriate in clinical scenarios with high-stress concentrations, while IPS E.max remains a preferred choice for cases prioritizing superior aesthetics. Specifically, the differences in stress transfer between IPS E.max and Lava Ultimate and the impact of increasing diastema width were successfully modeled using FEA, providing valuable guidance for clinical applications.

These findings highlight that material selection and tooth preparation are critical in stress distribution across restorations. Specifically, IPS E.max’s high elastic modulus results in more significant stress accumulation, increasing the risk of crack formation in thin restorations. In contrast, Lava Ultimate’s lower elastic modulus allows for broader stress distribution, reducing fracture risk. This emphasizes that material choice is crucial for the long-term success of thin ceramic veneer restorations.

### Comparison between full crown and laminate veneer restorations

Dejak et al. [16] demonstrated that full crowns effectively distribute stresses due to their larger surface area and homogeneous coverage, maintaining maximum stress levels within a safe range. In contrast, with their thinner structures, laminate veneers generate higher stress concentrations under vertical loads. Consistent with these findings, the present study observed that laminate veneers exhibited higher stress values (166.88 MPa) in the lateral incisor under a vertical load of 250 N. Such elevated stress levels indicate a potential risk of fracture. However, laminate veneers’ minimally invasive nature and excellent aesthetic outcomes make them desirable for anterior restorations. Full crowns, on the other hand, demonstrated superior stress distribution by spreading forces across a broader surface area, enhancing restoration durability. These findings are supported by Lin et al. [17], who emphasized the role of restoration design and material properties in managing stress distribution in ceramic restorations.

However, these findings depend not only on restoration type but also on masticatory dynamics. For instance, while laminate veneers offer the advantage of minimal preparation, they may fail over time due to inadequate adhesion or material fatigue. Conversely, full crowns distribute forces better due to their broader contact area but pose a risk of excessive tooth structure loss due to aggressive preparation. From a clinical perspective, these results suggest that while a more invasive approach may enhance the biomechanical advantages of a restoration, it must be balanced with the need to preserve tooth structure.

### Stress distribution in lateral teeth

Jiang et al. [14] highlighted that the narrow root structure and inclined position of lateral teeth often result in higher stress levels under vertical loading, challenging the long-term durability of restorations. Similarly, this study observed that lateral teeth experienced the highest stress concentrations under vertical loads, emphasizing the importance of accounting for anatomical characteristics during restoration planning to optimize outcomes and reduce fracture risk.

### Effects of IPS e.max and lava ultimate on dentin

Zheng et al. [18] investigated the biomechanical behavior of endocrown restorations with different CAD-CAM materials, highlighting that materials with high elastic moduli reduce stress levels in dentin by efficiently transferring forces. In this study, IPS E.max restorations exhibited a maximum dentin stress of 45.92 MPa, well below the reported fracture strength of dentin (97–150 MPa), indicating its safety in preserving dentin integrity. Conversely, Lava Ultimate restorations exhibited a higher maximum dentin stress of 65.74 MPa. Although these values remain within acceptable limits, they may contribute to long-term dentin fatigue or wear.

### Clinical implications and future research

The findings of this study emphasize the critical role of material selection in determining restoration success. IPS E.max, with its superior aesthetic properties and lower stress accumulation in dentin, is an ideal choice for anterior restorations where aesthetics are paramount. With its flexible structure and even stress distribution, Lava Ultimate is better suited for clinical scenarios involving high-stress concentrations. Additionally, restoration planning for lateral teeth requires careful evaluation of anatomical characteristics and loading conditions to optimize clinical outcomes.

More importantly, this study provides valuable insights into the biomechanical behavior of restorations used in diastema closure. However, it does not directly evaluate how these materials respond under dynamic loading conditions. Fundamental mastication forces differ from static analysis, as additional biomechanical factors such as lateral movements, bruxism, and sudden trauma must also be considered. Therefore, validating FEA results through future clinical and in vitro studies will provide more accurate predictions for patient-based applications.

This study’s findings suggest that FEA analysis can reliably predict stress distribution in restorations using CAD-CAM materials. FEA modeling is essential for identifying differences between IPS E.max and Lava Ultimate materials. Similarly, the impact of diastema width on restoration stress levels has been modeled, aiding clinicians in making more informed, case-specific decisions [14]. However, further mechanical and clinical research is necessary to translate these findings into clinical practice fully.

This study utilized finite element analysis (FEA) to evaluate stress distribution under static loading conditions. However, certain limitations must be acknowledged, including the assumption of homogeneous material properties and the inability to replicate dynamic clinical loading conditions. Since FEA analyses assume that materials are homogeneous and isotropic, they may not fully capture the natural variations in biological tissues. [19] Additionally, this study was conducted under static loading conditions, meaning that factors such as dynamic chewing forces, thermal fluctuations, and patient-specific dental morphology were not considered.

To overcome these limitations, future studies should incorporate patient-specific 3D scans, dynamic loading scenarios, and hybrid experimental-analytical approaches. Further research should also examine the performance of various CAD-CAM materials under clinically realistic conditions to provide a more comprehensive understanding of their behavior.

## Conclusion

- Due to its high elastic modulus, IPS E.max results in more significant stress accumulation, whereas Lava Ultimate distributes stress more evenly, thereby reducing localized stress intensity.
- Due to their thin structure, laminate veneers generate higher stress concentrations, while full crowns distribute stress more effectively across a broader contact area.
- 2 mm diastemas exhibited higher stress accumulation compared to 1 mm diastemas.
- IPS E.max reduces stress on dentin, offering protective benefits, while Lava Ultimate generates lower stress accumulation within the restoration itself.
- The highest stress levels were observed in lateral teeth due to their anatomical characteristics.
- IPS E.max suits cases with aesthetic requirements, whereas Lava Ultimate is a more durable option under high-stress conditions.
- While the finite element analysis (FEA) method effectively evaluates stress distribution, it cannot fully replicate dynamic clinical loading conditions.
- Future studies should investigate dynamic loading scenarios and the long-term performance of different CAD-CAM materials.

## Data Availability

No datasets were generated or analysed during the current study.
