# Correction to: Stress distribution in the closure of anterior maxillary diastemas using different restorative approaches: a finite element analysis

**DOI:** 10.1007/s00784-025-06302-8

**Published:** 2025-04-08

**Authors:** Kevser Karakaya, Rahime Zeynep Erdem

**Affiliations:** 1 Department of Prosthodontics, Faculty of Dentistry, Afyonkarahisar Health Sciences University, Afyonkarahisar, Turkey; 2 Department of Restorative, Faculty of Dentistry, Afyonkarahisar Health Sciences University, Afyonkarahisar, Turkey

**Correction to: Clinical Oral Investigations**

10.1007/s00784-025-06258-9

In the published paper, the institutional affiliation for the first author, Kevser Karakaya, was mistakenly listed as both the 1st institution (Department of Prosthodontics, Faculty of Dentistry, Afyonkarahisar Health Sciences University) and the 3rd institution (Department of Prosthodontics, School of Dentistry, Afyonkarahisar Health Sciences University, Guvenevler Neighborhood, Ismet Inonu Street, Afyonkarahisar, Turkey). These two institutional affiliations represent the same department, and only the 1st institution information should be valid. The 3rd institutional affiliation was erroneously included and merely represents the detailed address of the same institution.

The original article has been corrected.

